# Could sonic delivery of bulk-fill resins improve the bond strength and cure depth in extended size class I cavities?

**DOI:** 10.4317/jced.57310

**Published:** 2020-12-01

**Authors:** Karla-Janilee-de Souza Penha, Ana-Ferreira Souza, Marina-Jansen dos Santos, Lauber-José dos Santos-Almeida Júnior, Rudys-Rodolfo-De Jesus Tavarez, Leily-Macedo Firoozmand

**Affiliations:** 1 DDS, MSc, PhD student, Department of Dentistry, Federal University of Maranhão (UFMA), São Luís, Maranhão, Brazil; 2 MSc student, University of Campinas (UNICAMP), Piracicaba, SP, Brazil; 3 DDS, Federal University of Maranhão (UFMA), São Luís, Maranhão, Brazil; 4 DDS, MSc, PhD, Professor, Department of Post-Graduation Ceuma University (UNICEUMA), São Luis Maranhão, Brazil; 5 DDS, MSc, PhD, Professor, Department of Dentistry, Federal University of Maranhão (UFMA), São Luís, Maranhão, Brazil

## Abstract

**Background:**

The implementation of restorative procedures that guarantee success and optimize clinical time is the target of investigations in Restorative Dentistry. This study aimed to analyze the influence of sonic insertion of bulk-fill (BF) and conventional (C) resin composites on the microtensile bond-strength (µ-TBS) and cure depth (CD) of large and deep class I restorations.

**Material and Methods:**

Fifty-six healthy human premolars were selected and occlusal cavities (4 x 4 x 3 mm; factor C = 5) were prepared. TC - Tetric N-Ceram (BF), SF - SonicFill (BF), and Z350 - Filtek Z350 XT (C) composite resins were used to restore the cavities, using sonic (S) and non-sonic (NS) insertion techniques. A group restored with conventional incremental insertion (I) using Z350 XT resin was performed serving as a control. Teeth were prepared for microtensile bond-strength test (µ-TBS). And also, restoration depths of 1 and 4 mm were measured with an automatic microhardness indenter (50 g -15 s) to determine the CD. Results were evaluated using ANOVA, Scheffe, and Games-Howel posthoc test (α = 0.05).

**Results:**

Types of resins and insertion techniques present statistical differences for µ-TBS and CD (*p* ≤ 0.001). The µ-TBS was higher respectively for the groups SF > TC > Z350; however, the sonic insertion for SF and Z350 (I) did not present significant differences in µ-TBS. Higher microhardness values were observed on the surface (1mm). At a depth of 4 mm Z350 (I)> SF(S)> SF(NS)> TC(S/NS)> Z350(S/NS) (*p*< 0.001). Pearson’s Correlation of bond strength and base micro-hardness was significant (*p* ≤ 0.001), strong, and positive (0.955).

**Conclusions:**

The influence of sonic insertion is material dependent, influenced only the microhardness of the SonicFill resin and did not interfere with the bond strength and cure depth of other bulk fill and conventional resin composite.

** Key words:**Composite resins, dentin, hardness tests, tensile strength, Bulk-fill resins, sonic insertion.

## Introduction

The good aesthetic results and ease handling are aspects that make the resin composites a widely used material for direct restorations. The incremental stratification technique is recommended for reducing the tension caused by the polymerization shrinkage stress and adequate cure (1), by the greater conversion of the monomers into polymers is achieved in increments with a thickness less than 2mm (2).

The stratified insertion technique is established because, besides guaranteeing physical and mechanical properties, it reduces resin cytotoxicity (3). Clinically this technique also helps to reduce the marginal infiltration, cuspal deflection, and postoperative sensitivity (4). However, for large cavities, this technique requires considerable clinical time, in addition to risks of contamination between the resin layers and the inclusion of bubbles (5).

The idea of using materials that allow the application of a single increment is mainly aimed at simplifying clinical procedures and reducing working time (6). The main advantage of bulk resins is the insertion of material up to 4 or 5 mm in large class I and II cavities. This kind of resin composites has two viscosities: flowable (low viscosity), which is recommended as a base restorative material, and should be covered by a conventional resin for an anatomic sculping technique, and regular viscosity, which already allows for final anatomic sculpture (7).

The sonic technology was introduced to facilitate the handling and bulk insertion of up to 5 mm of resin, thus reducing the clinical time (8). The sonic energy has been proposed for increasing the fluidity of the resin composite and consequently allowing better adaptation to cavity preparation (9).

During the restoration of large cavities, an important issue to be considered is polymerization shrinkage stress that is generated by bonding more than 2 walls of the cavity preparation. This factor influences on the cure of the material and bond strength of resin composite to the dental substrate (10). Thus, the influence of sonic energy on the quality of restorations with a high cavitation configuration factor (C factor) should be investigated. While bond strength is an indicator of the retention capacity of restorative material to cavity preparation (11), microhardness indirectly influences the degree of conversion/ cure depth, and consequently, the clinical performance of resin restorations (12).

Due to the challenges inherent to the insertion of resin in large and deep cavities, and the possible benefits of the use of sonic energy stimulate investigations to verify its influence on the mechanical properties of resins. The advantage of sonic technique in relation to conventional techniques would optimize the insertion of composite resins, stimulating and justifying its use.

Thus, this study aimed to evaluate *in vitro* the bond strength and cure depth of bulk-fill composite resins using the sonic or non-sonic technique, and observe the correlation between these factors. The null hypothesis tested was that there is no influence of insertion techniques (sonic and non-sonic) of resin composites in the bond strength and the cure depth of conventional and bulk-fill resin composites.

## Material and Methods

-Teeth selection 

This study had the followed procedures in accordance with the ethical standards of the Research Ethics Committee – UFMA, São Luis (n.1.572.367) and with the Helsinki Declaration of 1975, as revised in 1983.

After performing the pilot test, the sample size was calculated considering an alpha level (α) of 5%, and eight teeth were randomly selected for each of the seven experimental groups. Thus, 56 extracted caries-free human premolars were selected and immersed in 0.2% thymol for a period not exceeding three months after extraction. They were cleaned with curettes to remove periodontal tissue debris, and prophylaxis was performed using pumice stone, water, and a Robinson brush.

-Cavity Preparations

Class I cavities were prepared using cylindrical diamond burs with rounded tips KG #3145 (KG Sorensen Ind, and Com, Ltda., Cotia, SP, Brazil), obtaining standard class I cavities 4mm deep (4x4x3) (13) resulting in high C factor (C factor=5). The dimensions of the cavity were measured using a digital calipter (Mitutoyo Sulamericana Ltda., Suzano, SP, Brazil), accepting a variability of ± 0.1 mm.

Five teeth per diamond bur were prepared by the same operator using a high-speed turbine and constant cooling.

-Restorative procedures and experimental groups

After cavity preparation, the enamel and dentin were conditioned for 30 and 15 s using 37% phosphoric acid (Condac 37, FGM, Joinville, SC, Brazil), respectively. The adhesive system Single Bond 2 (3M ESPE, St, Paul, MN, USA) was actively applied for 15 s and light-cured for 10 s using a LED curing light (Bluephase, Ivoclar Vivadent, Germany, 1200 mW/cm2).

The cavities were restored using the resin composites (n=16 each): TC - bulk-fill resin Tetric N-Ceram (Vivadent), SF - bulk-fill resin SonicFill and Z350 - conventional resin Filtek Z350 XT, and each group was subdivided into two subgroups using different insertion technique (n=8); sonic (S) and non-sonic (NS) (Table 1).

**Table 1:**
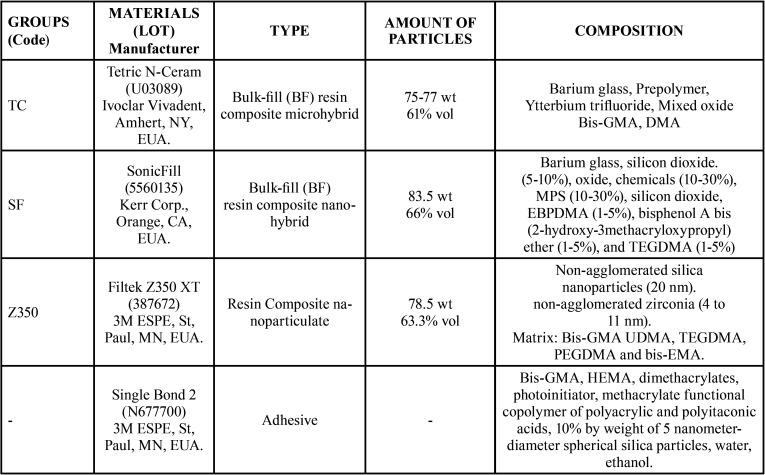
Groups and materials employed.

The counter-angle of sonic activation (Kavo Kerr, Joinville-SC, Brazil) was used, coupled with a micromotor, under speed 4, which allowed the insertion of the material at intermediate speed after the activation of the pedal. Single-dose capsules with composite resin were positioned in the counter-angle to allow its use. The composite resins Tetric N-Ceram and Filtek Z350 XT were positioned inside of empty capsules to facilitate their insertion into the cavity using the sonic technique. After filling each prepared cavity, photopolymerization was performed for 40 seconds from the occlusal surface.

Non-sonic insertion was conducted using a resin insertion spatula (Titaneo, Indusbello, Londrina - PR, Brazil) and the bulk insertion of the resins followed by slight vertical pressure. Photoactivation was conducted as described above.

To compare the behavior of the techniques employed, restorations were also performed with the Filtek Z350XT conventional resin (n=8) using the incremental technique whose results are already established in the literature, serving as a positive control. Three increments were placed in the cavity using a metal resin insertion spatula: two increments obliquely converging towards the center (joining a surrounding wall and a back wall) and followed by light curing of the occlusal surface, and finally by a horizontal increment to complete the restoration (14).

Each increment was cured for 20 s. The samples were stored in distilled water at 37 ± 1 ºC for 24 h.

-Microtensile bond strength test (µ-TBS)

The teeth were individually fixed on a precision cutting machine (IsoMet 1000, Buehler, Lake Bluff, IL, USA) with a diamond wheel. The teeth were perpendicularly sectioned in the buccolingual and mesiodistal directions to obtain specimens with approximately 1 mm² thickness in serial cuts. The sections were performed to obtain samples with resin composite and the dentin wall with dentinal tubules arranged perpendicular to the restoration.

Subsequently, the samples were fixed at their ends with a cyanoacrylate-based adhesive to the microtensile device, which was coupled to a universal testing machine (INSTRON Equipamentos e Sistemas de Ensaio Ltda., São José dos Pinhais, PR, Brazil). A speed of 0.5 mm/min was used until fracture.

The bond strength values were obtained in kilogram-force (Kgf), and the force required to fracture the specimens in Mega Pascal (MPa) was calculated by dividing the bond strength values (Kgf) by the sample area (mm2).

-Evaluation of fracture types

After the microtensile tests and bond strength evaluation, the fractured surfaces were analyzed using a stereomicroscope coupled to a digital camera.

The type of fracture was classified as: a) adhesive fracture: resin/adhesive interface fracture; b) cohesive fracture: dentin or composite resin fracture; or c) mixed fracture: fracture involving resin and adhesive/dental structure (combination of fracture types).

-Cross-sectional microhardness evaluation

A bipartite matrix (4 x 4 mm) was used to prepare semi-cylindrical samples using the previously described resins (n=16); TC (BF), SF(BF), and Z350(C). Each resin was divided into subgroups, and sonic (S) and non-sonic (NS) insertion techniques were employed (n=8 for each experimental condition). Sonic insertion was conducted using a sonic counter-angle (Kavo Kerr) as previously described, while non-sonic insertion was performed using a non-stick spatula. A positive control group, using the incremental technique (I) for conventional resin Z350 (n=8), was made as a control.

A glass slide was positioned with light pressure on the surface of the matrix filled by the resin composites to avoid the inclusion of air bubbles during polymerization and as a result a flat and uniform surface was obtained. Light curing was performed by positioning the active tip of the light-curing apparatus for 40 s on the glass slide. The samples were then stored in distilled water at room temperature for 24 h in an oven at 36±1°C. After this period, the samples were embedded with chemically activated acrylic resin (CAAS) and were then finished and polished with 400, 600, 800, 1200, and 4000-grain sandpaper for 30 s in a 600-rpm polishing machine under constant cooling.

-Microhardness Analysis 

The properly identified samples were taken to a Digital Microhardness Tester (Future-Tech Corporation, Tokyo, Japan) equipped with a pyramid-shaped Vickers diamond, using 50 g for 15 s (15). The microhardness of the resin surface (1 mm) and base (4 mm depth) of the sample was measured using three indentations conducted with 1000 μm spacing, and the mean microhardness of each area was obtained.

-Statistical analysis

The mean and standard deviation were calculated for of all resin composites and techniques. Data were checked for normal distribution by the Levene’s test. The data collected from µ-TBS and microhardness were analyzed using two-way ANOVA and post-hoc multiple comparison Scheffe Games-Howell test post hoc comparison test.

The software (SPSS for windows Version 26, SPSS Inc., Chicago, IL, USA) was used for analysis. A level of significance of α=0.05 was explored in all statistical tests.

## Results

Bond Strength Analysis 

The mean and standard deviation of the bond strength of the analyzed groups are presented in Table 2.

**Table 2:**
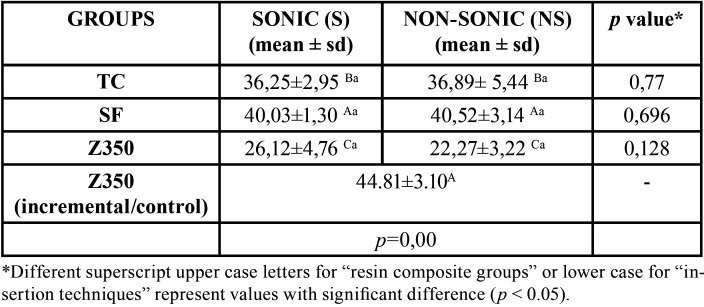
Mean values and standard deviation (sd) of bond strength for resin composites in different insertion techniques.

Sonic bulk insertion for both bulk-fill and conventional resins did not present significant difference in bond strength (Table 2). The SonicFill resin, irrespective of the insertion technique (sonic/non-sonic), had higher values of µ-TBS, and was not statistically different (*p*=0.142) from conventional incremental technique (resin Filtek Z350XT) (44.81±3.10) (positive control).

-Failure Mode Analysis of Debonded Specimens

**Figure 1:**
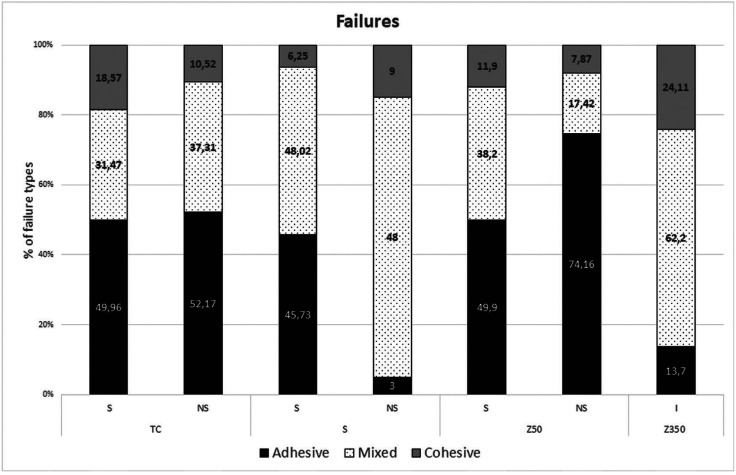
Distribution of fracture types.

Although there was a predominance of adhesive fractures in Tetric N-Ceram and Filtek Z350XT composite resins inserted with sonic and non-sonic insertion techniques. Mixed fractures were predominant observed in SonicFill composite resin and conventional Filtek Z350XT inserted incrementally (Fig. 1).

As presented in the previous studies, the pre-test failures were registered, but for the analyses of bond strength, the pre-test failures value were not considered (13,16). With the pre-test failure evaluation, it was observed a reduction of higher than 20% of viable samples for groups with lower values of µ-TBS groups (Z350 sonic/ non-sonic). Thus, a significant, positive, and moderate correlation between µ-TBS and the number of samples per tooth (without pre-test failure) was found. Pearson’s correlation (r=0.465; *p* ≤ 0.001).

-Cure depth analysis 

The mean and standard deviation values of the microhardness of the resins analyzed are presented in Table 3.

**Table 3:**
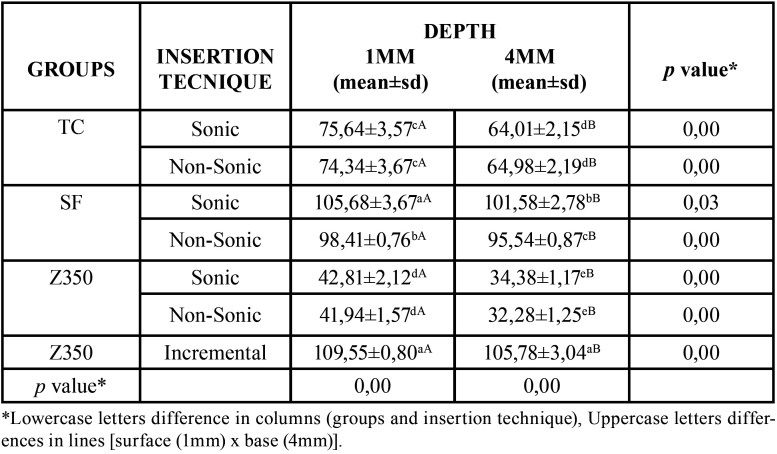
Mean values and standard deviations (sd) of vickers microhardness for all materials tested in different insertion techniques.

Both the insertion techniques and the cure depth showed significant statistical differences (*p* ≤ 0.001). The bulk-fill resins and the conventional resin had greater microhardness on the surface (1 mm) when compared with the restoration base (4 mm) (Table 3).

The sonic application of Sonicfill resin was the only one whose microhardness of the surface did not differ from incremental insertion of conventional resin. However, at the base of the restoration, the microhardness of all resins was lower than that of the incremental technique (conventional resin) (*p* ≤ 0.001).

Sonic insertion did not cause significant changes in the microhardness of the resins when compared with non-sonic insertion, except for the SonicFill resin (Table 3).

-Correlation between bond strength and microhardness of the restoration base (4 mm)

**Figure 2:**
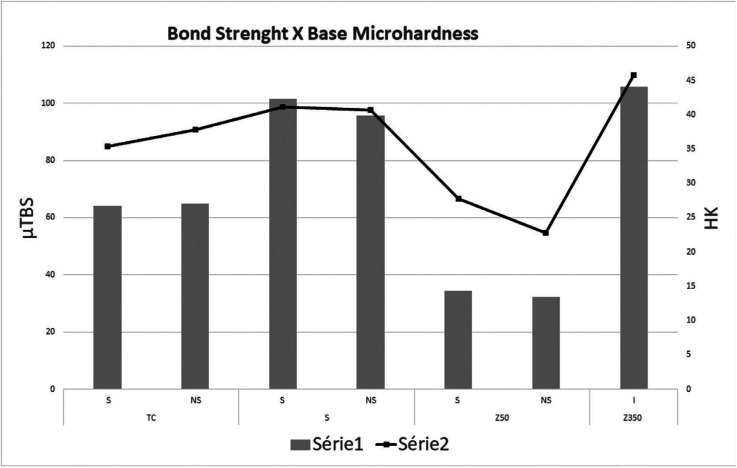
Pearson’s correlation between bond strength (µTBS) and base microhardness (HK) (*p* ≤ 0.001).

Pearson’s Correlation showed significant correlation between bond strength and base microhardness of the composite resins (*p* ≤ 0.001). This correlation is strong, and positive (0.955) (Fig. 2).

## Discussion

Reduction in technical sensitivity and clinical work time are factors that contribute to the wide acceptance of the use of bulk-fill resins for the restoration of large cavities. The results of the present study demonstrated that even though sonic insertions of Bulk-Fill and conventional resins do not change the bond strength, the sonic insertion of SonicFill resin results in high microhardness values, similar to the incremental insertion of conventional resin. Therefore, the null hypothesis was partially rejected.

The analysis of the bond strength of composite resins helps to predict the retention and longevity of restorations (17). The polymerization shrinkage and failure in the adhesive interface (18) can occur when the shrinkage stress exceeds the bond tension, thereby generating microgaps (approximately 10 to 20 μm) (19) compromising the bond strength. Microhardness, in contrast, is an indirect indicator of the degree of conversion of materials (10) and can provide information on wear resistance (20). Inefficient cure compromises the process of converting monomers into polymers (21). Residual monomers contribute to the reduction of the quality of physical-mechanical properties such as adhesion and chemical stability of the resin composite, being potentially toxic to pulp tissues (22). Very deep cavities with high C factor, such as those evaluated in this study, constitute a clinical situation that greatly impairs micromechanical properties (23). The incremental insertion of conventional resins in large cavities ensures more favorable physical-mechanical behavior (14,24), representing the “gold standard” restorative technique, as observed in the present study.

Bulk-fill resins inserted into 4 mm deep cavities showed bond strength and microhardness results that were significantly higher than those of the bulk insertion of conventional resin. One of the factors that contribute to the optimization of the cure of Bulk-fill composite resins is its higher translucency, associated with variations in monomeric composition and load particles (17,21,25). It is also suggested that the presence of plasticizer monomers to reduce shrinkage stress and polymerization modulators in the composition of these materials favors a lower polymerization shrinkage and a higher degree of conversion of monomers into polymers (15). These factors justify obtaining adequate bond strength and microhardness values, even when deep cavities were evaluated. However, these improvements notwithstanding, there are even lower microhardness values for bulk-fill resins when compared with conventional resins inserted by the incremental technique, corroborating the literature reviewed (25,26). This fact is also commonly attributed to the lower percentage of load particles of some bulk-fill resins (27). However, the composite resins used in this study do not present significant difference in the volume of inorganic particles, according to information from the manufacturer (Table 1). The differences observed can be justified by the interaction between the individual compounds of each material.

The values of the bond strength and microhardness obtained from sonically inserted SonicFill resin were higher than those of the other bulk-fill resins and similar to those of the incrementally inserted conventional resin. The sonic resin optimize their flow, improving the adaptability of the material to dental preparation (8,9), probably because of the high weight of barium glass and silicon dioxide, and the modifiers that react to sonic energy. In these materials, the sonic energy results in the reduction of the viscosity of the material by up to 87%, resulting in a reduction of up to 30% of the time of the restorative procedure (6). Furthermore, the load volume of this material may be responsible for minimizing the stress caused by the polymerization shrinkage process (28).

In contrast, Tetric N-Ceram resin showed intermediate values of µ-TBS and microhardness, both on the surface and at 4 mm depth, not being influenced by sonic energy. This composite resin had the smallest amount and volume of inorganic load. Unlike the other resins, it features Ivocerin® (Ivoclar Vivadent), a germanium-based photoinitiator, which according to the manufacturer, is significantly more light-reactive than camphorquinone, does not require the presence of an amine as a co-initiator and allows the material to polymerize faster and deeper. Moreover, Tetric N-Ceram has large pre-cured load particles (up to 50 μm), which would help in reducing the polymerization shrinkage (17). These factors ensured that the single insertion of SonicFill and Tetric N-Ceram resins had better results when compared with the single insertion of conventional composite resin. The insertion of conventional resin increments greater than 2 mm compromises polymerization, causing losses to the adhesive interface (18).

The reduced cohesive failure rate in composite resins may indicate a satisfactory performance of these materials, as the resin is not the weakest link in the restoration. A predominance of adhesive failures was observed, except for the SonicFill and Filtek Z350 XT incremental insert resin, which exhibited a higher percentage of mixed-type failures. Simultaneously, there was a gradual reduction in microhardness with an increase in depth in all the composite resins, except for the conventional resin, which was inserted incrementally. Polymerization depth depends on the amount of light energy that can pass through the material, considering the amount of light that is dispersed and absorbed (29). The extent of dispersion depends on the different refractive indexes of the components of the composite resins (21). There was a positive correlation between the reduction of microhardness (base) and the reduction of bond strength.

The technical limitations of this study refer to the performance of procedures in a laboratory environment, where restorations are not exposed to all physical, chemical and microbiological conditions that occur in the oral cavity. However, it is known that standardized laboratory studies are necessary in order to observe the behavior of the materials without bias. Based on the results obtained and considering the limitations of the present study, we can conclude that although it is suggested that sonic energy contributes to increased fluidity and better distribution of inorganic particles, it was only influential when the resin recommended by the manufacturer was used (SonicFill). This can be attributed to the amount and composition of the organic and inorganic matrix of this material. Furthermore, the limited access to information regarding the compositions of the investigated composite resins due to patent protection by the manufacturers was a limitation of the study.

## Conclusions

Although sonic insertion does not influence the bond strength of the bulk-fill and conventional resins tested, it significantly increases the superficial microhardness of the SonicFill resin.
